# Mitigating renal dysfunction in liver cirrhosis: Therapeutic role of ferrous sulphate, folic acid, and its co-administration

**DOI:** 10.1016/j.toxrep.2025.102026

**Published:** 2025-04-09

**Authors:** Debabrata Dash, Rishu Kumar Rai, Raj Kumar Koiri

**Affiliations:** Biochemistry Laboratory, Department of Zoology, Dr. Harisingh Gour Vishwavidyalaya (A Central University), Sagar, Madhya Pradesh 470003, India

**Keywords:** Liver cirrhosis, Kidney disease, Folic acid, Ferrous sulfate, Antioxidant pathway, Glycolytic pathway

## Abstract

The liver and kidneys are vital organs for detoxification and metabolic regulation. Regular consumption of alcohol and acetaminophen can cause liver cirrhosis. Cirrhosis increases oxidative stress in kidneys by disrupting the balance between reactive oxygen species (ROS) and antioxidants, leading to cell damage. Folic acid and ferrous sulfate are two anti-anemic drugs treat various diseases, have a high reactive oxygen radical quenching ability, resulting in protection against oxidative damage in aerobic cell. The aim of this study was to investigate mitigating renal dysfunction in liver cirrhosis and therapeutic potential effect of ferrous sulfate, folic acid and its co-administration caused by alcohol-acetaminophen induced liver cirrhosis. Animals were divided into six groups. Rats of normal control group received water and normal diet ad libitum; AC and LC group received 4.5 % alcohol and a combination of 4.5 % alcohol and acetaminophen (300 mg/kg bw) via drinking water respectively for seven days. After seven days, rats of LC+FS, LC+FA and LC+FS+FA received FS (5 mg/kg bw), FA (5 mg/kg bw) and FS+FA (5 mg/kg bw) respectively via drinking water for four weeks. Enzyme activity and protein expression were measured by semi-quantitative RT PCR and western blotting respectively. Results revealed that FS and FA treatment individually and together restored the antioxidant enzyme activity and the levels of glycolytic pathway towards normal which were affected due to liver cirrhosis. FS and FA are well known anti-anemic drugs and proved to be efficient agents for antioxidant and glycolytic enzymes alteration in liver cirrhosis. This novel approach could lead to new treatments.

## Highlights

• Liver cirrhosis affects kidney function via glycolysis, oxidative stress & SGOT, SGPT, urea, creatinine elevation.
• Alcohol & acetaminophen co-consumption severely harms liver & kidney health.
• Antioxidants & glycolytic enzymes are potential targets for treating cirrhosis-induced kidney dysfunction.
• Liver cirrhosis alters glycolysis via LDH, PDHE1α, HIF1α, PFKFB3 and GLUT1/2 contributing to renal fibrosis.
• Ferrous sulfate & folic acid therapy improves renal function, boosts antioxidants & restores glycolysis.

## Graphical Abstract

**Figure:**
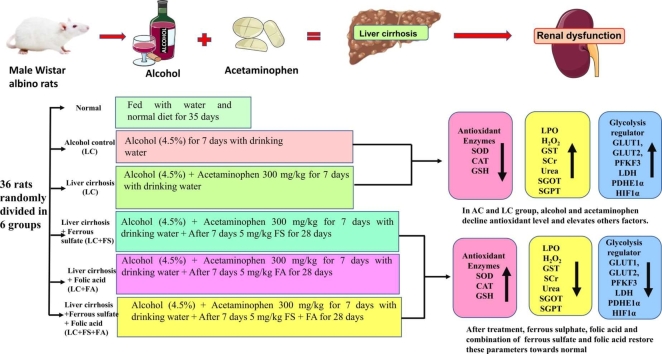

## 1 Introduction

The body's detoxification and metabolic processes, as well as maintenance of overall health, depend on the kidneys and liver. A key metabolic hub, the liver aids in the production of essential proteins and molecules as well as the removal of harmful substances from the body [1]. The kidneys simultaneously regulate fluid balance, waste elimination, and electrolytes through filtration and reabsorption mechanisms. Any alterations to the liver's structure, damage to it, or impairment of its functioning can result in a variety of health issues, including problems with the kidneys [2]. Modern medicine still faces challenges in managing liver disease [3]. Liver diseases are reported to cause for over 20,000 fatalities annually [4].

About 850 million people worldwide suffer from renal disease, with vulnerable populations being disproportionately affected. This includes 13.3 million cases of acute kidney injury (AKI) and 700 million cases of chronic kidney disease (CKD), primarily in low- and middle-income nations. With a 33 % increase in CKD cases between 1990 and 2017, the issue is getting worse. Early identification and screening programs are crucial for preventing this, particularly in low- and middle-income nations where the need is greatest [5]. Although kidney health can be impacted by a variety of factors, we will concentrate on the effects of liver cirrhosis.

Liver cirrhosis is a widespread condition characterized by fibrosis, nodular transformation, and abnormal structural changes, leading to various complication and poor outcomes [6]. Fatty liver disease, autoimmune diseases, and genetic metabolic disorder are among the causes of liver cirrhosis. Portal hypertension, liver dysfunction, and extrahepatic complication impacting several organs are all outcome of cirrhosis. In addition to the liver, cirrhosis also affects the heart, brain, lungs, intestines, kidneys, and immune system [7]. Abnormal hemodynamics, such as sympathetic activity, vasodilatation, and portal hypertension, contribute renal failure in cirrhosis. Renal insufficiency is the hallmark of hepatorenal syndrome (HRS), a dangerous complication of severe cirrhosis [8].

By interfering with physiological processes such as glycolytic and antioxidant pathways, liver cirrhosis damages kidney function by causing oxidative stress, increasing the production of free radicals, and cellular damage. Antioxidants are essential for preserving equilibrium [9]. Increased glucose flow into the kidneys, lactate formation, and glycolytic activity are all consequences of liver cirrhosis, which impacts the kidneys' energy metabolism and primarily depends on glycolytic pathways for energy production [10].

A common recreational drug, alcohol can damage the liver over time, resulting in alcoholic liver disease (ALD), which includes cirrhosis, hepatitis, and fatty liver. Alcohol metabolism in the liver involves alcohol dehydrogenase (ADH) and cytochrome P450 2E1 (CYP2E1), producing acetaldehyde, a toxic compound that forms adduct with cellular proteins and DNA, impairing function and promoting inflammation and fibrosis. Alcohol metabolism also generates reactive oxygen species (ROS), leading to oxidative stress and damage to lipids, proteins, and DNA. Furthermore, chronic alcohol consumption induces liver inflammation by activating Kupffer cells, which release pro-inflammatory cytokines, such as TNF-α and IL-6. Additionally, alcohol-induced oxidative stress and inflammation stimulate hepatic stellate cells (HSCs) to transform into myofibroblasts, which produce excessive extracellular matrix proteins, leading to fibrosis and ultimately cirrhosis [11]. Common painkiller acetaminophen is frequently used to treat hangover symptoms, but excessive dosage can result in nephrotoxicity and hepatotoxicity. At therapeutic doses, acetaminophen (APAP) is primarily metabolized by phase II conjugating enzymes, UDP-glucuronosyltransferase (UGT) and sulfotransferase (SULT), forming nontoxic compounds excreted in urine. A minor fraction is excreted unchanged. Approximately 5–9 % of APAP is metabolized by cytochrome P450 enzymes, mainly CYP2E1, generating the reactive intermediate N-acetyl-p-benzoquinone imine (NAPQI). Normally, NAPQI is detoxified by conjugation with glutathione (GSH). However, in overdose scenarios, excessive NAPQI depletes GSH, leading to covalent binding to cellular proteins, mitochondrial oxidative stress, and dysfunction, ultimately resulting in hepatocyte necrosis [12], [13]. This hepatic dysfunction can impair renal perfusion, leading to acute kidney injury (AKI) and chronic renal disease. In other words, cirrhosis and portal hypertension cause a neurohormonal cascade that leads to hepatorenal syndrome (HRS). This cascade induces vasodilation, which triggers compensatory mechanisms, resulting in renal vasoconstriction, hypoperfusion, and failure [2].

Intracellular enzymes called SGOT and SGPT are mostly located in the liver, but they are also found in trace amounts in other tissues such skeletal muscle and cardiac muscle [14], [15]. Serum albumin levels and liver enzymes like aspartate aminotransferase (AST/SGOT) and alanine aminotransferase (ALT/SGPT) are two laboratory indicators that can be used to evaluate liver damage in alcohol-acetaminophen-induced liver cirrhosis [16]. Impaired kidney function has also been confirmed by elevated blood urea nitrogen and creatinine levels [17].

A frequent side effect of liver cirrhosis, anemia can result from a number of causes and, by generating oxidative stress, can also induce kidney damage and chronic renal disease [18], [19]. It is essential to diagnose and treat these conditions as soon as possible. Antiviral medications, steroids, and vaccinations used to treat liver diseases might have negative side effects, particularly if taken for extended periods of time [20]. As a result, scientists are searching for alternative solutions to hepatic issues. Ferrous sulfate and folic acid are two examples of antioxidant supplements that can lessen the damage caused by oxidative stress. Two well-known anti-anemic medications that can address problems brought on by anemia are folic acid and ferrous sulfate. Ferrous sulfate is a form of iron used medicinally to treat and prevent iron deficiency anemia [21], and folic acid, also known as vitamin B9, is the synthetic form of folate found in fortified foods and supplements because it cannot be produced by mammals and must be obtained through diet [22], [23]. Developing treatment approaches to prevent or treat these disorders requires an understanding of the control of enzymes involved in the glycolytic process, the function of players involving the antioxidant system (SOD, CAT, GSH, H_2_O_2_, LPO), and biochemical parameters including SGOT, SGPT, urea, and creatinine. Therefore, the current study was created to assess the therapeutic potential of ferrous sulfate, folic acid, and their co-administration against alcohol-acetaminophen-induced liver cirrhosis in rats as well as their ability to mitigate renal impairment.

The present study aims to evaluate the therapeutic potential of ferrous sulfate, folic acid, and their co-administration in mitigating renal dysfunction associated with alcohol-acetaminophen-induced liver cirrhosis in rats. This study specifically investigates the biochemical parameters related to oxidative stress, glycolytic pathways, and renal function, including SGOT, SGPT, urea, and creatinine levels. Through the evaluation of oxidative and glycolytic factors as well as certain biochemical data, this study explores the relationship between liver cirrhosis and kidney cell injury. This integrated approach offers a comprehensive understanding of the connection between liver disease and renal health. The study of how acetaminophen and alcohol together impact liver and kidney function is a relatively recent area of research. While most studies focus on independently that is either on kidney or liver. This study fills a knowledge gap by investigating the cumulative or synergistic effects of alcohol and acetaminophen on organ damage. Folic acid and ferrous sulfate alone or in combination may be novel candidates for use as therapeutic medicines for renal damage brought on by liver cirrhosis. These agents are widely recognized for their benefits in other contexts, but their application here is new.

## 2 Materials and methods

### 2.1 Chemicals

Acetaminophen, ferrous sulphate, folic acid was procured from Cipla Pvt. Ltd. (India). Polyclonal antibody PDHE1α was procured from Santa Cruz Pvt. Ltd. HRP conjugated secondary antibody was procured from Merck Millipore Pvt. Ltd. ECL (Enhanced Chemiluminescence) substrate and Nitrocellulose membrane were purchased from BioRad Laboratories (India) Pvt. Ltd. Other chemicals until and unless specified were of analytical grade and purchased from HiMedia Laboratories, Pvt. Ltd. in Mumbai, India.

### 2.2 Experimental animals and treatment

A total of thirty-six male Wistar albino rats weighing 150 ± 25 g were randomly divided into six different groups. Rats were kept at the animal facility under optimum laboratory conditions, with proper supply of standard food and water *ad libitum*. All animal treatments were carried out in accordance with the requirements and approval of Institutional Animal Ethics Committee's (No. 379/CPCSEA/IAEC-2021/008). Food and water *ad libitum* was given to the rats in the normal control group (N). Alcohol control (AC) and the rats in the liver cirrhosis (LC) as well as liver cirrhosis rats treated with ferrous sulphate (LC+FS), folic acid (LC+FA) and a combination of ferrous sulfate and folic acid (LC+FS+FA) group were given 4.5 % alcohol and a combination of 4.5 % alcohol and acetaminophen (300 mg/kg bw) respectively in their drinking water for one week. After one week, rats in the ferrous sulphate (LC+FS) group were administered ferrous sulphate (5 mg/kg bw) through their drinking water, folic acid (LC+FA) group were administered folic acid (5 mg/kg bw) through their drinking water and a combination of ferrous sulfate and folic acid (LC+FS+FA) group were administered ferrous sulfate and folic acid (5 mg/kg bw) for four weeks.

### 2.3 Preparation of kidney homogenate and serum collection

Four euthanized rats (Pentobarbitone sodium, 50 mg/k.g. bw, i.p.) from each group were sacrificed by cervical dislocation and their kidneys were removed after the course of treatment. After being instantly cleaned with ice-cold phosphate buffer saline (pH 7.4), tissues were frozen and preserved at −80^◦^C until needed for a biochemical investigation. After homogenizing 10 % of the kidneys in 20 mM Tris buffer (pH 7.4) that had been frozen on ice, the supernatant was extracted by centrifuging the mixture at 16,000 x g for 45 min. For blood-based studies, blood was collected in heparin-free tubes from three to four rats in each group. Collected blood was allowed to clot at room temperature and centrifuged at × 1000 g for 20 min. Straw-colored serum was collected and frozen until required. Protein concentration in kidney extract was determined by the Lowry method using bovine serum albumin as standard [24].

### 2.4 Analysis of serum glutamate oxaloacetate transaminase (SGOT) and serum glutamate pyruvate transaminase (SGPT)

The activity of SGOT and SGPT was measured in accordance with manufacturer's instructions using a commercially available kit from ERBA Diagnostics Mannheim, Germany [25]. In order to conduct SGOT and SGPT, 200 mmol L-Aspartate, 545 U/L MDH, 909 U/L LDH, 0.18 mmol/L NADH (yeast), 12 mmol/L 2-Oxoglutarate, 80 mmol/L Tris buffer, pH 7.8, and 5.0 mmol/L EDTA were the working reagents for SGOT, and 500 mmol/L L-Alanine, 1820 U/L LDH, 12 mmol/L 2-Oxoglutarate, and 0.20 mmol/L NADH (yeast), Tris buffer (pH 7.6) were added to the cuvette for SGPT. After thoroughly mixing everything inside the cuvette, it was incubated for five minutes at 37^°^C. For 5 min, a drop in absorbance was seen at 340 nm when compared to distilled water as a standard.

### 2.5 Determination of urea level

As previously mentioned, [26], urea was measured using a commercially available kit from ERBA Diagnostics Mannheim, Germany. A urea reagent containing urease, glutamate dehydrogenase, α-ketoglutarate, and NADH was combined with a suitably diluted serum. When urease is present, urea is hydrolyzed to produce ammonia. Ammonia reacts with α-ketoglutarate to create L-glutamate and NAD when glutamate dehydrogenase and NADH are present. The amount of urea was determined by using a UV spectrophotometer to measure the change in absorbance at 340 nm for one minute at intervals of 20 seconds. The results were represented as milligrams per milliliter.

### 2.6 Determination of creatinine level

The creatinine level was tested using the commercial kit from ERBA Diagnostics Mannheim, Germany, using the previously mentioned protocol [27]. Serum was appropriately diluted and introduced to the creatinine reagent (sodium hydroxide and picric acid). The Jaffe's reaction occurs when creatinine and alkaline picrate combine to generate an orange-yellow color. The produced orange-yellow color's absorbance, which was measured at 505 nm for 5 min at intervals of 60 seconds, is directly correlated with the amount of creatinine. mg/dl was used to express the results.

### 2.7 Measurement of enzymatic activity antioxidants

#### 2.7.1 Superoxide dismutase

Using a slightly optimized methodology from Beauchamp and Fridovich 1971 [28], superoxide dismutase (SOD) activity was measured by photochemical inhibition of nitro blue tetrazolium chloride (NBT) reduction. kidney homogenate cytosolic fractions were combined with a 1 ml SOD assay cocktail combination that contained 12 mM L-methionine, 70 μm NBT, 3 μm EDTA, and 0.1 mM potassium phosphate buffer (pH 7.8). To start the reaction, 30 μl of 0.2 mM riboflavin and 20 μl of kidney supernatant were mixed with the cocktail mixture. The reaction mixture-containing test tubes were incubated during the day at RT. The reaction mixture tubes were incubated in the dark and at room temperature. The same reaction mixture was kept in the dark as a blank. Test tubes with reaction mixture free of kidney extract were used as controls and were incubated in both light and dark settings. A comparison was made between test tubes that contained the reaction mixture with enzyme extract and test tubes that contained the reaction mixture alone at 560 nm. The absorbance difference between the light and dark test tubes was used to estimate the amount of enzyme required to provide a 50 % inhibition of NBT reduction/min as a SOD unit and the results were expressed as units/mg protein.

#### 2.7.2 Catalase

We measured the catalase (CAT) activity according to the previously published protocol, with a few optimizations [29], [30]. The assay mixture contained 1 milliliter and comprised 0.05 M phosphate buffer (pH 7.0), 0.003 % H_2_O_2_, the decrease in absorbance was measured at 240 nm for 3 min at an interval of 30 seconds and the results were expressed as units/mg protein.

#### 2.7.3 Glutathione

The total glutathione assay was performed on the kidney sample in compliance with a previously described methodology [31]. 30 μl of kidney supernatant was mixed with 1 M Tris-Cl, pH 8.0, and 2 mM 5,5′-dithiobis-(2-nitrobenzoic acid) (DTNB). The reaction mixture colored yellow after 15 min of incubation at 37°C, and absorbance at 412 nm was measured. GSH concentrations were expressed as μM/mg protein.

### 2.8 Measurement of enzymatic activity of GST

GST activity was measured using the same approach as formerly described, with a few minor modifications in which 1-chloro-2, 4-dinitrobenzene (CDNB) and reduced glutathione (GSH) were used as reaction substrates [32]. In order to conduct this experiment, 1 ml assay of a cocktail containing 980 µl of PBS (pH 6.5), 100 mM CDNB and 100 mM glutathione were added. The reaction was initiated following the addition of 10 µl of kidney extract to the cocktail and a surge in optical density was measured at 340 nm for 5 min. The results were expressed in µM/mg protein.

### 2.9 Measurement of lipid peroxidation

Lipid peroxidation was measured by using a previously described method [33], [34]. Utilizing this method, two milligrams of thiobarbituric acid and one milligram of malonaldehyde (MDA) are combined in an acidic solution to produce tri-methionine, a pink substance with a maximum absorbance at 548 nm. After adding the necessary amount of enzyme extract to test tubes containing 1 ml of Tris maleate buffer, the tubes were incubated in water at 37°C for half an hour. TBA reagent (1.5 ml) was then added. The substance in the test tube was heated in boiling water for ten minutes and then allowed to cool. Before adding the pyridine/n-butanol solution at a 3:1 (v/v) ratio, the solution was allowed to cool. Next, 1 N NaOH was added to the test tubes and mixed to stop the reaction. Next the sample was allowed to stand for ten minutes and the absorbance at 548 nm was measured.

### 2.10 Measurement of hydrogen peroxide (H_2_O_2_)

With a few modest modifications, the method previously described was utilized to determine the hydrogen peroxide content [34]. Subsequently, each test tube was filled with 20 μl of kidney supernatant, 5 % dichromate, 0.01 M phosphate buffer (pH 7.0), and glacial acetic acid solution (1:3). Every sample was set in a bath of boiling water and allowed to boil for 10 min. The optical density at 570 nm was measured after cooling. A standard curve was used to calculate the H_2_O_2_ concentration, and the findings are reported as µM/mg protein.

### 2.11 Measurement of enzymatic activity of LDH

LDH activity was determined spectrophotometrically using Kornberg's (1955) method and as previously reported [35], [36]. Briefly, a 1 ml reaction mixture contains 3 mM NADH, 20 mM Tris-Cl (pH 7.4), 1 mM sodium pyruvate, and diluted tissue extract. Following the mixing of the reaction mixture and tissue extract, the reaction was initiated, and a drop in OD at 340 nm was noted per minute for 5 min. Values were represented as unit/mg protein.

### 2.12 Analysis of SOD, catalase, GST and LDH by non-denaturing PAGE

#### 2.12.1 Superoxide dismutase

For SOD, kidney extract containing 160 µg protein was loaded on a 12 % native gel and electrophoresis was carried out at 4ºC. Following electrophoresis, the gel was stained with a substrate-specific solution containing 2.5 mM NBT, 28 µM riboflavin, and 28 mM TEMED. The gel was incubated in the staining solution for 30 min in darkness, followed by exposure to white light until clear bands indicative of SOD activity became visible against a dark blue background [37]. The SOD specific PAGE followed by specific staining was carried out three times and the relative densitometric results were presented as mean ± standard deviation.

#### 2.12.2 Catalase

For catalase, kidney extract containing 160 µg proteins was loaded on 8 % native gel and electrophoresis was carried out at 4ºC. Following electrophoresis, the gels were treated with 0.003 % H_2_O_2_ and incubated in darkness for a period of 10 min. Hydrogen peroxide is converted into water and oxygen by catalase enzymes in the regions of the gel where it is localized. Next, two separate solutions were prepared in individual tubes: a 2 % potassium ferricyanide solution and a 2 % ferric chloride solution. These two solutions were then mixed together to create a potassium ferricyanide reagent solution The gel was treated with the potassium ferricyanide reagent until achromatic bands became visible against a dark green background. Catalase prohibited the reduction of potassium ferricyanide to ferrocyanide, which reacts with ferric chloride to produce green precipitate, by eliminating hydrogen peroxide. Following the appearance of catalase bands on a dark green background, the gel was promptly rinsed with distilled water, and the bands were then quantified using gel densitometry software [37]. The PAGE for catalase specificity was done at least three times and the relative densitometric results were presented as mean ± standard deviation.

#### 2.12.3 Glutathione S-transferase (GST)

For GST, kidney extract containing 160 µg protein was loaded on 10 % native gel and electrophoresis was carried out at 4ºC [37]. After electrophoresis, the gel was allowed to equilibrate in a 0.1 M potassium phosphate buffer solution (pH 6.5) for a period of 10 min. After that, the gel was incubated for 10 min at room temperature with a reaction mixture comprising 4.5 mM GSH, 1 mM CDNB and 1 mM NBT dissolved in 0.1 M potassium phosphate buffer (pH 6.5). The gel was further incubated in the dark for an additional 10 minutes at room temperature in a solution containing 3 mM PMS in 0.1 M Tris-HCl buffer (pH 9.6). Achromatic bands corresponding to GST activity became visible against a blue background. GST specific PAGE was carried out at least three times and relative densitometric results were represented as mean ± standard deviation.

#### 2.12.4 Analysis of LDH isozymes

12 % non-denaturing polyacrylamide gel electrophoresis (PAGE) for LDH containing 160 μg proteins was carried out using a technique previously described, with some optimizations as outlined in previous work [38]. The gel was treated with a staining solution specifically designed for LDH activity, which contained 100 mM Lithium lactate, 125 mM Tris-Cl buffer (pH 7.4), 100 mM NaCl, 0.5 mM MgCl_2_, 0.25 mg/ml NBT, 1 mg/ml NAD and 0.025 mg/ml PMS after electrophoresis was completed. Distinct dark black purple formazan bands, corresponding to various LDH isozymes, were visible due to their unique migration patterns in the gel under the influence of the electric field. The LDH specific PAGE was carried out three times and the relative densitometric results were presented as mean ±standard deviation.

### 2.13 Western blotting

Western blot analysis of 160 μg protein samples was done on 10 % SDS-PAGE, followed by overnight electro transfer onto nitrocellulose or PVDF membrane, as previously described [30]. After confirming protein transfer with Ponceau S stain, the membrane was rinsed with PBS. Membranes were blocked in PBS containing 5 % skimmed milk for 2 h at room temperature. Blocked membranes were treated with a 1:1000 dilution of primary antibody at 4°C overnight. Blots were washed three times in PBST (phosphate buffer saline with 0.1 % Tween 20) at 5 minute intervals. This was followed by a 2 h incubation on a rocker with a 1:2000 dilution of secondary antibody (anti-rabbit coupled with horseradish peroxidase). The membrane was rinsed four times with PBST, each for five minutes. The bands on the membrane were identified with an ECL substrate, and the protein bands were measured using gel densitometry software.

### 2.14 Semi-quantitative reverse-transcription polymerase chain reaction

Total RNA was isolated from liver tissue using the TRI reagent and following the manufacturer's instructions. According to manufacturer’s instructions full length first strand cDNA was synthesized from RNA template using random hexamer primer from iScript Select cDNA synthesis kit from Bio-Rad laboratories. PCR reaction mixture contained 1 x PCR buffer, 10 mM dNTPs, 10 pmol/μl primer and 1 unit of Taq polymerase. Specific primers for the rat genes were used: GLUT1 (Forward 5′-TGGCCAAGGACACACGAATACTGA-3׳, Reverse 3′-TGGAAGAGACAGGAATGGGCG-AAT-5′); GLUT2 (Forward 5׳-TAGTCAGATTGCTGGCCTCAGCTT-3׳, Reverse 3׳-TTGCCCTGACTTCCTCTTCCAAC-5׳); HIF1α (Forward 5׳-GGTGCTAACAGATGATGGTG-3׳, Reverse 3׳-CTGGCTCATAACCCATCAAC-5׳); PFKFB3 (Forward 5′-CGCAATAGTGTCACCCCACT-3′, Reverse 3′-TCCCTAGCAAAGGTTGTCCG-5′); and GAPDH (Forward 5׳-AGCCCAGAACATCATCCCTG-3׳, Reverse 3׳-CACCACCTTCTTGATGTCATC-5׳). The 6X DNA loading dye was combined with the amplified PCR product and loaded onto a 2 % reducing agarose gel that was run in 1X Tris-Acetate EDTA buffer (TAE), pH 8.5. The gel was quantified using gel densitometry software and viewed using a gel documentation system (UVP Chem Studio 315 Imaging System, Upland, CA, USA) [30].

### 2.15 Statistical analysis

The experimental data were presented as mean ± standard deviation. The student's *t*-test was used to determine the level of significance between the control and experimental groups. p < 0.05 was considered significant.

## 3 Results

### 3.1 Ferrous sulfate, folic acid and its coadministration treatment significantly reduces SGOT, SGPT, urea level, and SCr level in kidney during alcohol-acetaminophen induced liver cirrhosis

**Fig. 1:**
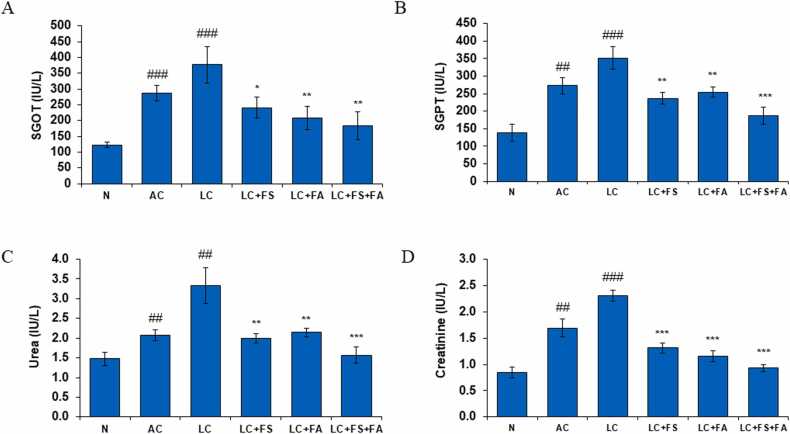
(A) Serum glutamate oxaloacetate transaminase, (A) Serum glutamate pyruvate transaminase, (C) Blood urea and (D) serum creatinine level in normal, alcohol control, liver cirrhosis (LC), treatment of ferrous sulphate (LC + FS), treatment of folic acid (LC + FA), treatment of ferrous sulphate and folic acid (LC + FS + FA). Value represent mean ± SD. ^##^p < 0.01, ^###^p < 0.001 (normal vs. AC or LC). *p < 0.05, ^**^p < 0.01, ^***^p < 0.001 (LC vs. LC + FS or LC + FA or LC + FS + FA).

In the present investigation, our results show that the activity of serum marker like serum glutamate oxaloacetate transaminase (SGOT), serum glutamate pyruvate transaminase (SGPT), SCr and urea level was considerably increased in alcohol-acetaminophen induced LC rats as compared to normal control. However, folic acid and ferrous sulfate and its combination, significantly decreased the SGOT, SGPT, SCr and urea level and brought them toward the normal level, thereby suggesting the beneficial effect our treatment drugs in kidneys of LC rats (Fig. 1).

### 3.2 Ferrous sulfate, folic acid and is coadministration treatment significantly increases antioxidant level (SOD, CAT, GSH) in kidney during alcohol-acetaminophen induced liver cirrhosis

#### 3.2.1 Superoxide dismutase (SOD)

**Fig. 2:**
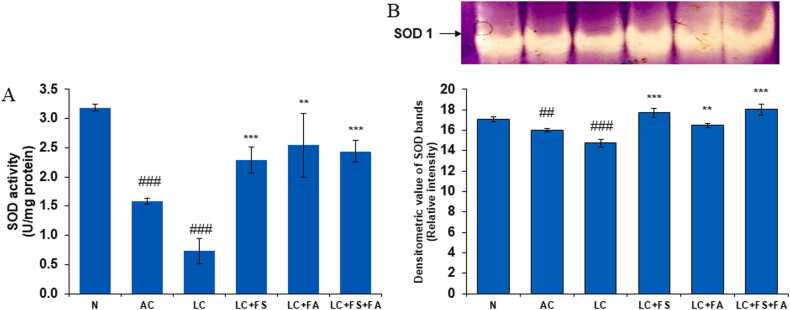
(A) Superoxide dismutase (SOD) level in the kidney of normal, alcohol control, liver cirrhosis, treatment of ferrous sulphate (LC + FS), treatment of folic acid (LC + FA), treatment of ferrous sulphate and folic acid (LC + FS + FA). SOD activity, (B) gel image is representative of SOD activity, performed on 12 % Native-PAGE with 160 µg kidney supernatant loaded in each lane followed by substrate development of SOD bands. In the lower panel, relative densiometric values of SOD bands from control and experimental groups have been presented as mean ± SD from three PAGE repeats. Value represent mean ± SD. ^##^p < 0.01, ^###^p < 0.001 (normal vs. AC or LC). ^**^p < 0.01, ^***^p < 0.001 (LC vs. LC + FS or LC + FA or LC + FS + FA).

Reactive oxygen species (ROS) build up in kidney tissues as a result of many pathways that liver cirrhosis activates. This leads to oxidative stress and damage to cellular constituents such as proteins, lipids, and DNA. Together, superoxide dismutase and catalase are the most important antioxidants in the human body, assisting in reducing the harmful effects of oxidants. The first antioxidant enzyme in defense is superoxide dismutase (SOD). It Neutralize superoxide radicals (O_2_^-^) i.e., SOD converts superoxide into hydrogen peroxide (H_2_O_2_) and oxygen (O_2_), thereby reducing the harmful effects of superoxide. In the present study, compared with the normal control group, the alcohol control group and the liver cirrhosis group exhibited considerably decreased SOD activity. Treatment of LC rats with ferrous sulfate, folic acid and combination of both restored SOD activity towards normal and thus, minimizing its adverse renal damaging effects (Fig. 2).

#### 3.2.2 Catalase (CAT)

**Fig. 3:**
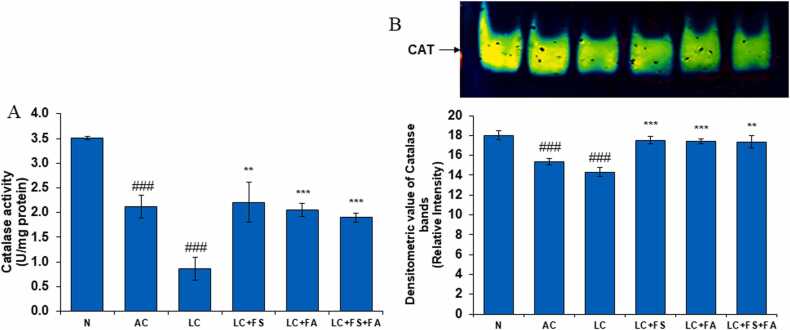
(A) Catalase (CAT) level in the kidney of normal, alcohol control, liver cirrhosis, treatment of ferrous sulphate (LC + FS), treatment of folic acid (LC + FA), treatment of ferrous sulphate and folic acid (LC + FS + FA). CAT activity, (B) gel image is representative of catalase activity, performed on 8 % Native-PAGE with 160 µg kidney supernatant loaded in each lane followed by substrate development of CAT bands. In the lower panel, relative densiometric values of CAT bands from control and experimental groups have been presented as mean ± SD from three PAGE repeats. Value represent mean ± SD. ^##^p < 0.01, ^###^p < 0.001 (normal vs. AC or LC). ^**^p < 0.01, ^***^p < 0.001 (LC vs. LC + FS or LC + FA or LC + FS + FA).

The initial line of defense against oxidative stress is provided by downstream enzymes such as CAT and antioxidant enzymes such as SOD. As the activity of these enzymes decreases, the redox state of the cells changes. In the antioxidant enzyme system after SOD; catalase and GPx are two enzymes which are responsible for the detoxification of hydrogen peroxide. In the present investigation, there was significant decline in the level of catalase in alcohol control and liver cirrhosis rats. Decline in the activity of catalase is expected to result in the accumulation of harmful H_2_O_2_, which can cause DNA damage and initiate renal disease. Post treatment of LC rats with folic acid and ferrous sulfate restored catalase activity of kidney towards normal and thus, could exert antiproliferative effect by detoxifying hydrogen peroxide, thereby, minimizing its adverse renal damaging effects (Fig. 3).

### 3.3 Administration of chronic alcoholism and acetaminophen-induced liver cirrhosis significantly increase the level of GST in kidney

**Fig. 4:**
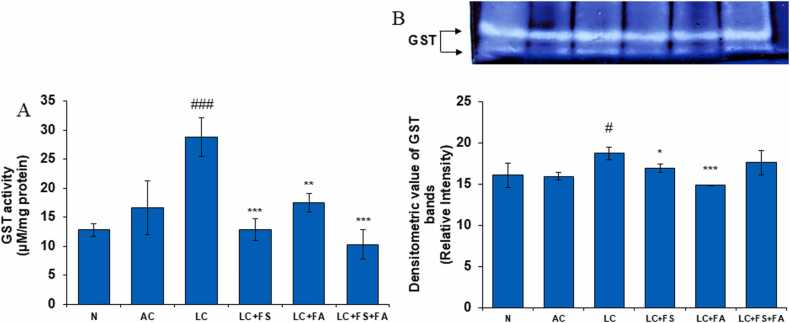
(A) GST level in the kidney of normal, alcohol control, liver cirrhosis, treatment of ferrous sulphate (LC + FS), treatment of folic acid (LC + FA), treatment of ferrous sulphate and folic acid (LC + FS + FA). GST activity, (B) gel image is representative of GST activity, performed on 10 % Native-PAGE with 160 µg kidney supernatant loaded in each lane followed by substrate development of GST bands. In the lower panel, relative densiometric values of GST bands from control and experimental groups have been presented as mean ± SD from three PAGE repeats. Value represent mean ± SD. ^#^p < 0.05, ^###^p < 0.001 (normal vs. AC or LC). *p < 0.05, ^**^p < 0.01, ^***^p < 0.001 (LC vs. LC + FS or LC + FA or LC + FS + FA).

Glutathione S-transferases (GST) are a family of enzymes that are known to catalyze the conjugation of reduced glutathione to a number of substrates ultimately resulting in detoxification. It plays a critical role in cellular detoxification against xenobiotics and toxic compounds as well as against oxidative stress. Glutathione S-transferase (GST) enzymes are involved in the detoxification of reactive metabolites of renal disease. We could observe that during alcohol-acetaminophen induced there was a significant decline in majority of antioxidant enzyme with a concomitant increase in lipid peroxidation. In line with this observation, there was significant increase in the activity of GST in kidney of AC and LC rats. Treatment of LC rats with folic and ferrous sulfate alone and combination of both led to a significant decrease in the activity of GST, thereby suggesting efficient detoxification and promotion of anti-renal damage activity (Fig. 4).

### 3.4 Ferrous sulfate, folic acid and is coadministration imparts renal protection by declining the level of lipid peroxidation and H_2_O_2_ in LC rats

**Fig. 5:**
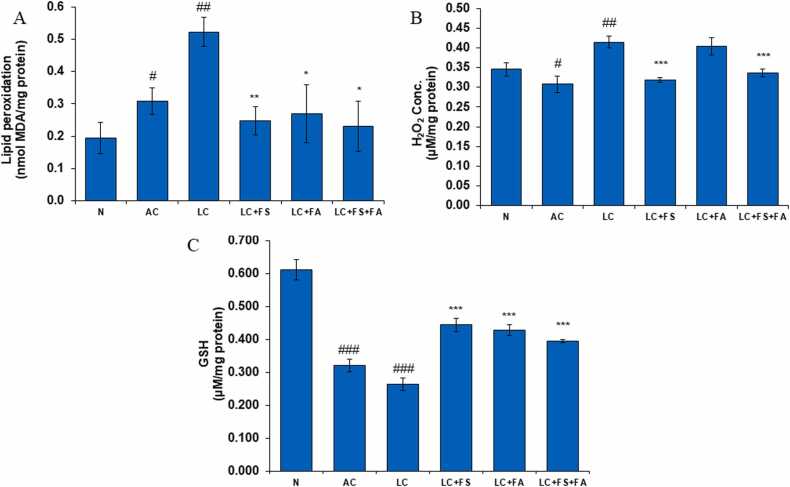
(A) Lipid peroxidation level (B) H_2_O_2_ level (C) GSH level, in the kidney of normal, alcohol control, liver cirrhosis, treatment of ferrous sulphate (LC + FS), treatment of folic acid (LC + FA), treatment of ferrous sulphate and folic acid (LC + FS + FA) enzyme activity. Value represent mean ± SD. ^#^p < 0.05, ^##^p < 0.01, ^###^p < 0.001 (normal vs. AC or LC). *p < 0.05, ^**^p < 0.01, ^***^p < 0.001 (LC vs. LC + FS or LC + FA or LC + FS + FA).

Lower cellular free radical scavenging abilities and greater free radical formation during LC metabolism may be connected to the presence of free radical-induced membrane lipids (MDA) and hydrogen peroxide (H_2_O_2_) in kidney tissue. The current study revealed that the levels of lipid peroxidation (LPO) and hydrogen peroxide (H_2_O_2_) in the kidney significantly increased in AC and LC groups. Treatment of LC rats with folic acid and ferrous sulfate and its combination restored LPO and H_2_O_2_ level towards normal (Fig. 5A, B).

### 3.5 Glutathione (GSH)

Glutathione has the ability to protect vital biological components from damage caused by peroxides, lipid peroxides, heavy metals, reactive oxygen species, and free radicals. Our study revealed that the GSH activity in the AC and LC groups was significantly lower than that in the normal control group. In treatment group GSH activity significantly higher than AC and LC groups. Our drugs restored GSH of kidney towards normal (Fig. 5C).

### 3.6 Ferrous sulfate, folic acid and is coadministration reduces lactate dehydrogenase and restore towards normal level in renal tissue

**Fig. 6:**
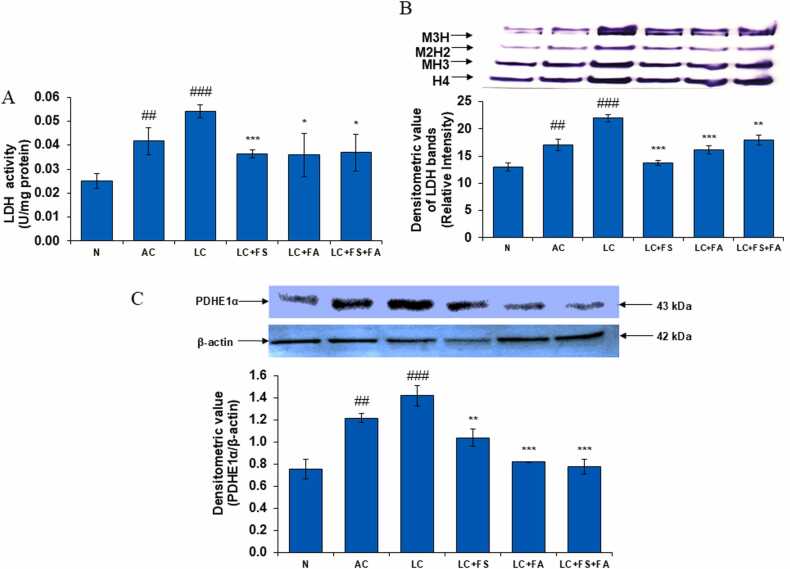
(A) LDH level in the kidney of normal, alcohol control, liver cirrhosis, treatment of ferrous sulphate (LC + FS), treatment of folic acid (LC + FA), treatment of ferrous sulphate and folic acid (LC + FS + FA). LDH activity, (B) gel image is representative of LDH activity, performed on 10 % Native-PAGE with 160 µg kidney supernatant loaded in each lane followed by substrate development of LDH bands. In the lower panel, relative densiometric values of LDH bands from control and experimental groups have been presented as mean ± SD from three PAGE repeats. (C) Shows protein expression of PDHE1α. Above figures represent western blot photograph with 160 µg protein in kidney, in each lane, presented with densitometric values of PDHE1α/β actin bands as mean ± SD from 3 western blot repeats. Value represent mean ± SD. ^##^p < 0.01, ^###^p < 0.001 (normal vs. AC or LC). *p < 0.05, ^**^p < 0.01, ^***^p < 0.001 (LC vs. LC + FS or LC + FA or LC + FS + FA).

Lactate dehydrogenase (LDH) is key enzyme of the glycolytic pathway that catalyzes the conversion of pyruvate to lactate & vice versa. Lactate dehydrogenase is a commonly used marker in toxicology and clinical chemistry for assessing damage to cells, tissues, and organs. In the present investigation, there was significant increase in LDH activity in alcohol-acetaminophen induced LC. LDH activity as well as in the activity of M4/M3H/M2H2 LDH isozymes and the significant increase in LDH activity was detected in the kidney of LC rats. Post treatment of LC rats with folic acid, ferrous sulfate and folic acid + ferrous sulfate led to a significant decline the activity of LDH, LDH isozymes and restored them towards normal level (Fig. 6A, B).

### 3.7 Ferrous sulfate, folic acid and is coadministration reduces PDHE1α in renal tissue during alcohol-acetaminophen induced liver cirrhosis

PDHE1α (Pyruvate Dehydrogenase E1) is an enzyme that plays a crucial role in cellular metabolism. It is a component of the Pyruvate Dehydrogenase Complex (PDC) or PDH, which is a crucial enzyme linking glycolysis to the citric acid cycle. PDH is responsible for converting pyruvate into acetyl-CoA. PDH controls glucose metabolism in kidney tissues, especially in the proximal tubules. The current study revealed that the protein expression of PDHE1α in the kidney significantly increased in alcohol control as well as liver cirrhosis group. Post treatment of LC rats with folic acid, ferrous sulfate and folic acid + ferrous sulfate led to a significant decline the expression of PDHE1α (Fig. 6C).

### 3.8 Alcohol-acetaminophen induced LC involves upregulation of HIF1α whereas ameliorative effect of ferrous sulfate, folic acid and is coadministration involves their inhibition

**Fig. 7:**
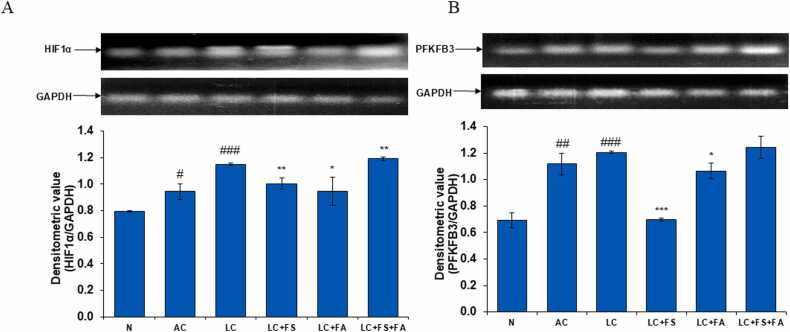
(A) HIF1α and (B) PFKFB3 mRNA expression in the kidney of normal, alcohol control, liver cirrhosis, treatment of ferrous sulphate (LC + FS), treatment of folic acid (LC + FA), treatment of ferrous sulphate and folic acid (LC + FS + FA). Above figure in (A) & (B) is a representative RT-PCR photograph for HIF1α and PFKFB3 with the densitometric values of HIF1α/GAPDH and PFKFB3/GAPDH mRNA presented as mean ± SD from three RT-PCR repeats respectively. Value represent mean ± SD. ^#^p < 0.05, ^##^p < 0.01, ^###^p < 0.001 (normal vs. AC or LC). *p < 0.05, ^**^p < 0.01, ^***^p < 0.001 (LC vs. LC + FS or LC + FA or LC + FS + FA).

Hypoxia inducible factors (HIFs) are critical to sense intratumoral oxygen tension and subsequently mediate the activation of hypoxia response, thus representing a potential kidney injury target. In the present investigation, we observed that there was a significant increase in the mRNA expression of HIF-1α in alcohol-acetaminophen induced LC. Although post treatment of LC rats with folic acid and ferrous sulfate led to a significant decline in the expression of HIF-1α in kidney of LC rats (Fig. 7A).

### 3.9 Alcohol-acetaminophen induced LC involves upregulation of PFKFB3 whereas ameliorative effect of ferrous sulfate, folic acid and is coadministration involves their inhibition

PFKFB3, a crucial glycolytic activator in renal fibrosis. Fructose-6-phosphate is converted by PFKFB3 to fructose-2,6-bisphosphate, which is a strong activator of phosphofructokinase-1 (PFK-1), an essential glycolysis enzyme. In the present investigation, we found that there was a noticeable increase in the mRNA expression of PFKFB3 in kidney of LC rats. However, a noticeable, decline in the mRNA expression of PFKFB3 was observed after post treatment with folic acid and ferrous sulfate and its combination, in alcohol-acetaminophen induced LC rats (Fig. 7B).

### 3.10 Ameliorative effect of ferrous sulfate, folic acid and is co-administration on glycolytic regulators i.e., glucose transporter1 (GLUT1, GLUT2) and PFKFB3 in alcohol-acetaminophen induced LC

**Fig. 8:**
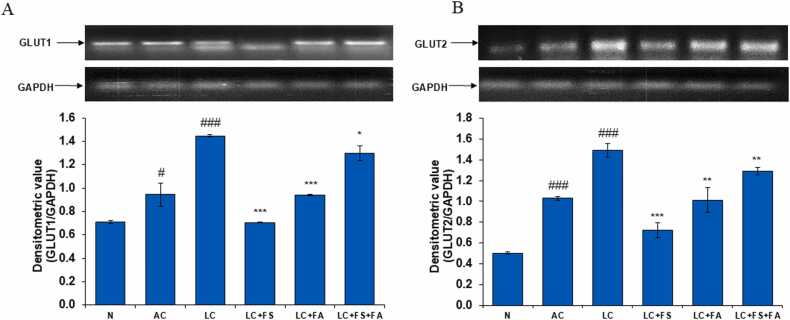
(A) GLUT1 and (B) GLUT2 mRNA expression in the kidney of normal, alcohol control, liver cirrhosis, treatment of ferrous sulphate (LC + FS), treatment of folic acid (LC + FA), treatment of ferrous sulphate and folic acid (LC + FS + FA). Above figure in (A) & (B) is a representative RT-PCR photograph for GLUT1 and GLUT2 with the densitometric values of GLUT1/GAPDH and GLUT2/GAPDH mRNA presented as mean ± SD from three RT-PCR repeats respectively. Value represent mean ± SD. ^#^p < 0.05, ^###^p < 0.001 (normal vs. AC or LC). *p < 0.05, ^**^p < 0.01, ^***^p < 0.001 (LC vs. LC + FS or LC + FA or LC + FS + FA).

GLUT1 and GLUT2 are glucose transporter proteins that play important roles in the kidney. GLUT1 facilitates glucose transport out of the cell into the bloodstream. GLUT2 facilitates glucose transport from the filtrate into the cell. Together, GLUT1 and GLUT2 work in tandem to regulate glucose reabsorption and metabolism in the kidney. In the present study, compared with the normal control group, the alcohol control group and the liver cirrhosis group exhibited considerably high GLUT1 and GLUT2 expression. Treatment of LC rats with folic acid and ferrous sulfate restored GLUT. Further, among three treatment Ferrous sulfate treat more effectively and thus, minimizing its adverse renal damaging effects (Fig. 8A, B).

## 4 Discussion

Excessive alcohol consumption damages multiple organs, particularly the liver, by altering metabolic pathways and inducing inflammation, ultimately leading to cirrhosis. The hepatotoxic effects of alcohol are mediated by the production of acetaldehyde through alcohol dehydrogenase, as well as the activation of hepatic stellate cells, oxidative stress, inflammation, and upregulation of cytochrome P450 2E1 (CYP2E1), collectively contributing to liver injury [39], [40]. All of these processes work together to cause liver cirrhosis, which highlights how crucial moderate alcohol consumption is to avoiding liver damage. Additionally, hepatocytes caused by consumption of excess alcohol and paracetamol releases this serum enzymes like SGOT and SGPT into the bloodstream. In our current investigation, SGOT and SGPT levels were noticeably higher in liver cirrhotic rats with alcohol-acetaminophen-induced liver cirrhosis (Fig. 1A-D), suggesting that alcohol-acetaminophen induces liver injury. Interestingly, our findings are consistent with a previous study [41], which assessed increases in SGOT and SGPT activities following hepatotoxic administration of CCl_4_, ethanol, and acetaminophen to rats. However, SGOT and SGPT levels significantly decreased (returned to normal) with the administration of ferrous sulfate, folic acid and a combination of both. This suggests that folic acid and ferrous sulfate may protect against the liver damage brought on by alcohol. Recent research supports our findings, showing that folic acid administration significantly reduces the activity of SGOT and SGPT in rats given ethanol [42]. Moreover, ferrous sulfate is used as iron supplementation and some studies suggest iron supplementation can ameliorate alcohol-induced liver damage in iron-deficient animals, highlighting the therapeutic potential of iron in reducing hepatic injury and promoting liver health [43].

Beyond the liver**,** liver cirrhosis also affects multiple organs, including the kidneys, heart, brain, lungs, intestines, and immune system [44]. Chronic kidney disease (CKD) or acute kidney injury (AKI) can result from untreated liver cirrhosis. Specifically, portal hypertension, vasoconstrictive hormone activation, arterial vasodilatation, systemic inflammation, and oxidative stress (ROS) are some of the factors that lead to renal failure. AKI associated with cirrhosis alters serum creatine levels [45]. Additionally, renal hypoperfusion brought on by cirrhosis-induced portal hypertension results in hypoxia and oxidative stress in kidney tissues due to ROS production [46]. A prevalent comorbidity in alcoholic liver cirrhosis is anemia, which increases the production of ROS and hypoxia, leading to oxidative stress and cellular damage [47]. Alcohol-induced anemia depletes iron, vitamin B12, and folic acid through malabsorption, malnourishment, and direct toxicity. As a result, renal hypoxia and damage occur due to a reduction in oxygen delivery to tissues [48]. Importantly, the ability of blood to carry oxygen is compromised by decreased hemoglobin content, which exacerbates hyperdynamic circulation and sympathetic activity that are crucial to the pathophysiology of Hepatorenal Syndrome (HRS) [47]. Thus, anemia is a major contributing factor to oxidative stress, renal hypoxia, and HRS in chronic liver disease.

From a biochemical perspective, oxidative stress contributes to renal damage in both acute and chronic kidney dysfunction. Lipid peroxyl radicals, superoxide, nitric oxide, and the extremely reactive hydroxyl radical are examples of reactive oxygen species (ROS), which are unstable, extremely reactive molecules with oxygen and unpaired electrons that can oxidize lipids, proteins, and DNA. Despite numerous reports of elevated ROS in human and animal models of kidney injury, the specific factors and mechanisms underlying ROS enhancement, signaling mechanisms, and impact on endogenous antioxidant systems remain uncleared.

To counteract oxidative stress, several antioxidants such as Glutathione (GSH), glutathione reductase (GR), catalase (CAT), glutathione peroxidase (GPx), and superoxide dismutase (SOD), that shield kidney cells from oxidative stress and damage caused by reactive oxygen species (ROS). Notably, the degree of kidney injury can be determined by the expression of these enzymes. In this regard, folic acid (Vitamin B9) reduces oxidative stress by reducing plasma homocysteine levels [49], while ferrous sulfate, which is used to treat iron deficiency anemia, enhances antioxidant activity by reducing the amounts of hydrogen peroxide and malondialdehyde [50].

Our study highlights that ferrous sulfate indirectly lessens oxidative stress in renal tissues by reducing anemia. Two criteria have been used in this case to evaluate the antioxidant enzymes: the quantitative assessment of the enzymatic activity in cell-free extracts and the amount of antioxidant enzymes ascertained by an in-gel test based on nondenaturing PAGE analysis. An in-gel-based enzyme detection method was created and used to ascertain the physiological significance at the cellular level in order to obtain a suitable profile of the enzymatic proteins and their active fractions [51]. A quantitative assessment of the enzyme activity was also carried out in order to validate the PAGE profile results.

By neutralizing superoxide radicals (O_2_-), superoxide degradation products (H_2_O_2_), and oxygen (O_2_), it lessens the harmful effects of superoxide. All three SOD isoforms SOD1, SOD2, and SOD3 are often found in the kidney and are anchored in the cytoplasm, extracellular space, and mitochondria by their heparin binding domain [52]. In the current study SOD activity was considerably lower in the alcohol control and liver cirrhosis groups than in the normal control group. These findings are in agreement with prior research showing that SOD levels are reduced in liver oxidative stress and histological changes induced by acetaminophen [53].

Importantly folic acid and ferrous sulfate treatment of LC rats reduced the harmful effects of oxidative stress in kidney bringing the SOD level back to normal.

In general, SOD activity is similar in the kidneys of mice, pigs, sheep, cows, rabbits, and humans [52]. SOD inhibition causes the kidneys to produce more ROS, which reduces medullary blood flow, glomerular filtration, and salt excretion. In rats, it may also result in hypertension [54]. Reduced SOD2 levels make the problem worse; in particular, CKD patients have lower SOD2 levels in their neutrophils under stress, which leads to an overabundance of ROS produced by malfunctioning neutrophils [55].

Similarly, Catalase is the second enzyme in the antioxidant pathway that breaks down hydrogen peroxide into oxygen and water. Thus, reduced catalase activity can exacerbate oxidative stress. Our native PAGE data show that the alcohol control and liver cirrhotic groups had lower catalase (CAT) activity than control groups. In response to stress or disease, catalase activity often increases to remove harmful H_2_O_2_. Our results align with a previous studies showing that LPS-induced catalase depletion exacerbates kidney injury in a mouse model of endotoxemia [56]. Reducing reactive oxygen species (ROS) and halting lipid peroxidation require catalase. Due to shortage of catalase reactive oxygen species (ROS) build up in the mitochondria, which hinders the mitochondria's capacity to function [57]. After receiving folic acid and ferrous sulfate treatment, catalase level in kidneys of LC rats returned to normal. This suggests that hydrogen peroxide may have an antiproliferative impact by detoxifying the gas and reducing its harmful effects on the kidneys.

Furthermore, Phase II detoxification enzymes known as glutathione S-transferases (GSTs) are important for the biotransformation or detoxification of both endogenous and exogenous toxins. It offers protection against oxidative stress. Glutathione (GSH) substitutes an easily removed group on the xenobiotic, avoiding the ensuing harmful reactions. These enzymes, known as GSTs, are found in the cytoplasm of cells and catalyze a variety of substitution activities [58]. In the current investigation, the alcohol-acetaminophen treated group showed higher levels of GST activity than the normal control group. When alcohol and acetaminophen are administered together, folic acid, ferrous sulfate, and their combined effects improve GST activity in LC rats. Their anti-anemic and antioxidant properties might be the reason.

One important intracellular antioxidant that protects against hydroperoxides and free radicals is GSH, a thiol. It usually exists in cells at concentrations between 1 and 10 mM. Because of its high electron-donating capacity (sulphydryl group), GSH has a significant reducing power. This ability is used to regulate a complicated thiol exchange system that is essential for eicosanoid metabolism, antioxidant defense, and xenobiotics [59]. Our results show a significant decrease in GSH levels, which could suggest that GSH facilitates the removal of cytosolic H_2_O_2_ and our findings are supported by some reports showing glutathione is also decreased in CKD patients [60] and a rat model of diabetic nephropathy [61], in addition to lower SOD and catalase levels. After receiving ferrous sulfate and folic acid, the GSH level was significantly higher than that of the AC and LC groups. Our findings are supported by similar outcomes observed when folic acid was given to treat renal impairment brought on by APAP [62].

Hydrogen peroxide (H_2_O_2_) and membrane lipids (MDA) may be found in renal tissue due to free radicals generated by higher metabolism in liver cirrhosis and a reduced ability of cells to scavenge free radicals (lower total thiol). Lipid peroxidation is an important indicator of oxidative stress. The present study discovered that administering alcohol and acetaminophen to Wistar rats resulted in a significant increase in lipid peroxidation (LPO) and hydrogen peroxide (H_2_O_2_). These findings are consistent with earlier research by varsha Rani, which found that dimethyl nitrosamine-induced acute renal failure is associated with a substantial rise in H_2_O_2_ levels [63]. A link between increased MDA levels and APAP toxicity was suggested by Dallak and associates [64]. As a result, hydrogen peroxide accumulates and can react with transition metals to produce dangerous and highly reactive species that have reactivities comparable to that of the hydroxyl radical. Following administration of folic acid and ferrous sulfate together, the LPO level of the LC rats was brought back to normal.

By using SCr and urea levels as biomarkers, kidney damage had been confirmed [65]. According to the current investigation, alcohol-acetaminophen-induced liver injury was indicated by the considerably increased serum creatinine and urea levels in LC rats with alcohol-acetaminophen-induced liver cirrhosis (Fig. 1C-D). After administration of ferrous sulfate, folic acid, or a combination of the both, there was a significant reduction in urea and creatinine levels, restored to normal levels. This implies that ferrous sulfate and folic acid may be protective against alcohol-induced liver cirrhosis (Fig. 1C-D).

Beyond oxidative stress, the pathophysiology of both acute and chronic kidney disease is significantly influenced by hypoxia, making it an appropriate target for therapeutic approaches. One of the most promising candidates for future oxygen-improving therapies appears to be HIF, the master switch of hypoxia adaptation responses. HIF is crucial for cellular adaptation to hypoxia, improving survival and tissue oxygenation while preventing negative effects on the kidney's supply and tolerance to hypoxia [66]. This adaptation leads to a decrease in cellular oxygen consumption and an increase in glycolysis [67]. Additionally, this metabolic alteration protects the cells by reducing the generation of ROS. By upregulating the glucose transporters GLUT1 and GLUT3, HIF activation increases the expression of enzymes involved in the glycolytic process and facilitates the absorption of glucose. When hypoxia is brief and temporary, this defense mechanism functions effectively [67], [68]. The expression of glycolytic enzymes like HK2, PKM2, PGK1, and LDHA1 is stimulated by HIF1α. By enhancing the glycolytic pathway, this allows cells to produce energy even in the presence of low oxygen levels [69]. Our result also stands with these cellular metabolic changes, which revealed increase level of HIF-1α in LC rats and ferrous sulfate effectively treat the disease. Also, level of GLUT1 and GLUT2 were significantly increase in LC groups. Which indicates upregulation in glycolysis.

The shift toward glycolysis was further supported by increased lactate dehydrogenase (LDH) activity, indicating enhanced anaerobic metabolism in renal tissues. Lactate dehydrogenase (LDH), a crucial part of the glycolytic pathway, catalyzes the conversion of pyruvate to lactate [70]. Glycolytic flux in kidney tissues, particularly in the proximal tubules, must be regulated by LDH [71]. Some previous studies have shown that acute kidney damage is also linked to a significant increase in LDH activity and levels, which causes anaerobic glycolysis and tissue degradation [72]. Glycolytic flux and tumor growth are also promoted by elevated LDH expression in kidney cancer [73]. Concurrent release of LDH from renal tissue confirms cellular damage and reinforces elevated urea levels, a hallmark of renal cell injury. Our study found that when folic acid, ferrous sulfate, and folic acid + ferrous sulfate were administered to LC rats, the expression of LDH dramatically dropped.

Another crucial metabolic alteration observed in this study was the dysregulation of pyruvate dehydrogenase E1-alpha (PDHE1α), a key enzyme linking glycolysis to the citric acid cycle. Pyruvate Dehydrogenase E1, or PDHE1α, is an enzyme that is essential to cellular metabolism. It is a part of the Pyruvate Dehydrogenase Complex (PDC), often known as PDH, an essential enzyme that connects the citric acid cycle with glycolysis. PDH regulates the metabolism of glucose in the kidney's tissues, particularly in the proximal tubules. Because of its high affinity for pyruvate, PDH guarantees efficient conversion to acetyl-CoA [74]. Our results showed that PDHE1α was significantly dysregulated, indicating that the rats in the LC and AL groups had higher levels of PDHE1α than the rats in the normal control group. PDH dysregulation aggravates kidney diseases, including diabetes and kidney cancer [73]. Furthermore, our study shows that when folic acid, ferrous sulfate, and combination of both folic acid and ferrous sulfate were given to LC rats, the expression of PDHE1α significantly dropped.

Again, Increased hexokinase 2 (HK2) expression in renal tissues of cirrhotic rats further supports the metabolic shift toward glycolysis. The enzyme hexokinase catalyzes the first step of glucose metabolism. In the current investigation, LC rats with alcohol-acetaminophen-induced liver cirrhosis had noticeably higher levels of hexokinase in their kidneys. According to previous research [75], increased levels of Hexokinase 2 (HK2) are associated with renal damage, that supports our results that alcohol and acetaminophen-induced liver cirrhosis damages the kidneys. When ferrous sulfate, folic acid, or both were administered to rats, HK2 levels significantly decreased and restore towards normal. This suggests that ferrous sulfate, folic acid, or both may be protecting against liver cirrhosis brought on by alcohol-acetaminophen.

Furthermore, another factor that is renal fibrosis, which results from the activation of renal fibroblasts, is a significant characteristic of chronic kidney diseases. The role of glycolysis in this process has been highlighted by recent studies. Yet, 6-phosphofructo-2-kinase/fructose-2,6-bisphosphatase 3 (PFKFB3), an essential glycolytic activator in renal fibrosis, is little understood. PFKFB3 transforms fructose-6-phosphate into fructose-2,6-bisphosphate, a potent activator of phosphofructokinase-1 (PFK-1), a crucial enzyme for glycolysis [76]. In the present investigation, PFKFB3 levels were markedly increased in the kidney of LC rats with alcohol-acetaminophen-induced liver cirrhosis. PFKFB3 has been shown to increase glycolytic flow, which raises fibroblast activation and contributes to the development of renal fibrosis [77]. The expression of PFKFB3 is significantly elevated in the renal tissues of mice following unilateral ureteral obstruction (UUO) or ischemia/reperfusion (I/R) injury, suggesting a critical role for PFKFB3 in the pathogenesis of kidney fibrosis [76]. After post-treatment with ferrous sulfate, folic acid, or both, there was a notable decrease in PFKFB3 levels towards normal. This suggests that ferrous sulfate, folic acid, or both may be protecting against liver cirrhosis brought on by alcohol-acetaminophen.

Overall, the study found that liver cirrhosis impairs antioxidant defenses, raises oxidative stress, and damages the kidneys. Through thorough data analysis, we found that acetaminophen and alcohol co-administration in rats had a synergistic effect that significantly increased oxidative stress, glycolytic enzyme activity, and HIF1α expression, as well as urea and serum creatinine levels, when compared to alcohol administration alone. This implies that combination of acetaminophen and alcohol may intensify nephrotoxicity and metabolic dysregulation, However, treatment with ferrous sulfate and folic acid enhances the body's capacity to detoxify, boosts antioxidant enzyme levels, and improves kidney function.

Although our study offers important new information about the protective benefits of folic acid (FA) and ferrous sulfate (FS) against liver and kidney damage caused by alcohol-acetaminophen, some limitations should be taken into account. First, a rat model was employed for the study, which may not accurately represent the intricacy of human pathophysiology while being a popular preclinical research tool. Second, there was a small sample size, which might have limited the findings' statistical strength and generalizability. Confirming these findings and evaluating the possible translational uses of FS and FA in human populations would require further research with bigger cohorts and clinical studies.

## 5 Conclusion

In conclusion, this study unequivocally demonstrates the negative impact of chronic alcoholism and acetaminophen-induced liver cirrhosis on kidney function, which is characterized by increased oxidative stress, disrupted glycolytic metabolism, ureal level, serum glutamate, and thus renal damage. Our data indicate that ferrous sulfate (FS) and folic acid (FA), both alone and combined, may have protective benefits via modifying oxidative stress, glycolytic metabolism, and important antioxidant defenses, potentially contributing to renal function preservation. The study sheds light on the involvement of HIF1α, GLUT1, GLUT2, PDHE1α, LDH, HK2, PFKFB3, and antioxidant enzymes in renal damage, and their prospective therapeutic targets. Although these results are encouraging, the study's limitations include its small sample size and dependence on an animal model. To confirm these results in clinical settings, examine the long-term effectiveness of FS and FA, and look into the underlying mechanisms in human populations, more research is needed. In order to determine the wider applicability of FS and FA in treating renal dysfunction linked to liver cirrhosis, future research should concentrate on clinical trials and mechanistic assessments. Even though this study offers insightful information about the liver-kidney relationship, it is crucial to consider the results in the context of preclinical research.

## CRediT authorship contribution statement

**Koiri Raj Kumar:** Writing – review & editing, Writing – original draft, Supervision, Resources, Project administration, Investigation, Funding acquisition, Formal analysis, Conceptualization. **Rai Rishu Kumar:** Writing – original draft, Visualization, Validation, Methodology, Investigation, Data curation. **Dash Debabrata:** Writing – original draft, Visualization, Validation, Methodology, Investigation, Formal analysis, Data curation, Conceptualization.

## Funding

This study was financially supported by a project from DST-SERB (CRG/2018/003780) and DBT-BUILDER (BT/INF/22/SP47619/2022), Govt. of India, sanctioned to RKK and the Indian Council of Medical Research (10.13039/501100001411ICMR), New Delhi, India (No.3/1/2(10)/CD/2022-NCD-II) to DD.

## Declaration of Competing Interest

The authors declare that they have no known competing financial interests or personal relationships that could have appeared to influence the work reported in this paper.

## Data Availability

Data will be made available on request.
