# Supplementary material for: Mitigating renal dysfunction in liver cirrhosis: Therapeutic role of ferrous sulphate, folic acid, and its co-administration
Source: Toxicol Rep. 2025 Apr 9;14:102026. doi: 10.1016/j.toxrep.2025.102026 (PMC12017912; doi:10.1016/j.toxrep.2025.102026)
Supplement: Supplementary file 1 — Supplementary material [file mmc1.docx]

| **Bonferroni’s multiple comparison test** | **t** | **Significant? P < 0.05?** | **Summary** |
| --- | --- | --- | --- |
| N vs AC | 10.9545 | Yes | *** |
| N vs LC | 7.5934 | Yes | *** |
| LC vs LC+FS | 3.5714 | Yes | * |
| LC vs LC+FA | 4.2989 | Yes | ** |
| LC vs LC+FS+FA | 4.6022 | Yes | ** |

SGOT (Fig. 1A)

SGPT (Fig. 1B)

| **Bonferroni’s multiple comparison test** | **t** | **Significant? P < 0.05?** | **Summary** |
| --- | --- | --- | --- |
| N vs AC | 6.7967 | Yes | ** |
| N vs LC | 9.2108 | Yes | *** |
| LC vs LC+FS | 5.5797 | Yes | ** |
| LC vs LC+FA | 4.7986 | Yes | ** |
| LC vs LC+FS+FA | 7.1617 | Yes | *** |

Urea (Fig. 1C)

| **Bonferroni’s multiple comparison test** | **t** | **Significant? P < 0.05?** | **Summary** |
| --- | --- | --- | --- |
| N vs AC | 4.6542 | Yes | ** |
| N vs LC | 6.6085 | Yes | ** |
| LC vs LC+FS | 4.9438 | Yes | ** |
| LC vs LC+FA | 4.3787 | Yes | ** |
| LC vs LC+FS+FA | 13.0754 | Yes | *** |

Creatinine (Fig. 1D)

| **Bonferroni’s multiple comparison test** | **t** | **Significant? P < 0.05?** | **Summary** |
| --- | --- | --- | --- |
| N vs AC | 7.4524 | Yes | *** |
| N vs LC | 17.6392 | Yes | *** |
| LC vs LC+FS | 12.0268 | Yes | *** |
| LC vs LC+FA | 13.8976 | Yes | *** |
| LC vs LC+FS+FA | 19.6061 | Yes | *** |

SOD enzyme activity (Fig. 2A)

| **Bonferroni’s multiple comparison test** | **t** | **Significant? P < 0.05?** | **Summary** |
| --- | --- | --- | --- |
| N vs AC | 35.91267 | Yes | *** |
| N vs LC | 18.68973 | Yes | *** |
| LC vs LC+FS | 8.531741 | Yes | *** |
| LC vs LC+FA | 5.316765 | Yes | ** |
| LC vs LC+FS+FA | 10.23281 | Yes | *** |

SOD in gel assay (Fig. 2B)

| **Bonferroni’s multiple comparison test** | **t** | **Significant? P < 0.05?** | **Summary** |
| --- | --- | --- | --- |
| N vs AC | 4.479 | Yes | ** |
| N vs LC | 7.366 | Yes | *** |
| LC vs LC+FS | 9.294 | Yes | *** |
| LC vs LC+FA | 5.892 | Yes | ** |
| LC vs LC+FS+FA | 7.641 | Yes | *** |

CAT enzyme activity (Fig. 3A)

| **Bonferroni’s multiple comparison test** | **t** | **Significant? P < 0.05?** | **Summary** |
| --- | --- | --- | --- |
| N vs AC | 10.062 | Yes | *** |
| N vs LC | 19.771 | Yes | *** |
| LC vs LC+FS | 5.075 | Yes | ** |
| LC vs LC+FA | 7.852 | Yes | *** |
| LC vs LC+FS+FA | 7.232 | Yes | *** |

CAT in gel assay (Fig. 3B)

| **Bonferroni’s multiple comparison test** | **t** | **Significant? P < 0.05?** | **Summary** |
| --- | --- | --- | --- |
| N vs AC | 5.432 | Yes | ** |
| N vs LC | 8.682 | Yes | *** |
| LC vs LC+FS | 9.750 | Yes | *** |
| LC vs LC+FA | 12.667 | Yes | *** |
| LC vs LC+FS+FA | 8.876 | Yes | *** |

GST enzyme activity (Fig. 4A)

| **Bonferroni’s multiple comparison test** | **t** | **Significant? P < 0.05?** | **Summary** |
| --- | --- | --- | --- |
| N vs AC | 1.40 | No | ns |
| N vs LC | 8.04 | Yes | *** |
| LC vs LC+FS | 7.31 | Yes | *** |
| LC vs LC+FA | 5.40 | Yes | ** |
| LC vs LC+FS+FA | 7.69 | Yes | *** |

GST in gel assay (Fig. 4B)

| **Bonferroni’s multiple comparison test** | **t** | **Significant? P < 0.05?** | **Summary** |
| --- | --- | --- | --- |
| N vs AC | 0.132 | No | ns |
| N vs LC | 2.582 | Yes | * |
| LC vs LC+FS | 2.482 | Yes | * |
| LC vs LC+FA | 6.474 | Yes | ** |
| LC vs LC+FS+FA | 1.209 | No | ns |

LPO level (Fig. 5A)

| **Bonferroni’s multiple comparison test** | **t** | **Significant? P < 0.05?** | **Summary** |
| --- | --- | --- | --- |
| N vs AC | 3.099 | Yes | * |
| N vs LC | 4.943 | Yes | ** |
| LC vs LC+FS | 4.406 | Yes | ** |
| LC vs LC+FA | 2.518 | Yes | * |
| LC vs LC+FS+FA | 3.254 | Yes | * |

H2O2 Level (Fig. 5B)

| **Bonferroni’s multiple comparison test** | **t** | **Significant? P < 0.05?** | **Summary** |
| --- | --- | --- | --- |
| N vs AC | 2.448 | Yes | * |
| N vs LC | 5.311 | Yes | ** |
| LC vs LC+FS | 10.401 | Yes | *** |
| LC vs LC+FA | 0.678 | No | ns |
| LC vs LC+FS+FA | 7.808 | Yes | *** |

GSH Level (Fig. 5C)

| **Bonferroni’s multiple comparison test** | **t** | **Significant? P < 0.05?** | **Summary** |
| --- | --- | --- | --- |
| N vs AC | 13.66 | Yes | *** |
| N vs LC | 16.31 | Yes | *** |
| LC vs LC+FS | 11.15 | Yes | *** |
| LC vs LC+FA | 11.37 | Yes | *** |
| LC vs LC+FS+FA | 11.72 | Yes | *** |

LDH enzyme activity (Fig. 6A)

| **Bonferroni’s multiple comparison test** | **t** | **Significant? P < 0.05?** | **Summary** |
| --- | --- | --- | --- |
| N vs AC | 4.438 | Yes | ** |
| N vs LC | 12.043 | Yes | *** |
| LC vs LC+FS | 9.230 | Yes | *** |
| LC vs LC+FA | 3.345 | Yes | * |
| LC vs LC+FS+FA | 3.634 | Yes | * |

LDH in gel assay (Fig. 6B)

| **Bonferroni’s multiple comparison test** | **t** | **Significant? P < 0.05?** | **Summary** |
| --- | --- | --- | --- |
| N vs AC | 5.056 | Yes | ** |
| N vs LC | 9.198 | Yes | *** |
| LC vs LC+FS | 9.832 | Yes | *** |
| LC vs LC+FA | 7.173 | Yes | *** |
| LC vs LC+FS+FA | 3.960 | Yes | ** |

PDHE-1-Alpha western (Fig. 6C)

| **Bonferroni’s multiple comparison test** | **t** | **Significant? P < 0.05?** | **Summary** |
| --- | --- | --- | --- |
| N vs AC | 6.407 | Yes | ** |
| N vs LC | 6.001 | Yes | ** |
| LC vs LC+FS | 3.532 | Yes | * |
| LC vs LC+FA | 5.669 | Yes | ** |
| LC vs LC+FS+FA | 5.186 | Yes | ** |

HIF-1-Alpha RT-PCR (Fig. 7A)

| **Bonferroni’s multiple comparison test** | **t** | **Significant? P < 0.05?** | **Summary** |
| --- | --- | --- | --- |
| N vs AC | 2.535 | Yes | * |
| N vs LC | 13.326 | Yes | *** |
| LC vs LC+FS | 2.787 | Yes | * |
| LC vs LC+FA | 2.954 | Yes | * |
| LC vs LC+FS+FA | 1.228 | No | ns |

PFKFB3 RT-PCR (Fig. 7B)

| **Bonferroni’s multiple comparison test** | **t** | **Significant? P < 0.05?** | **Summary** |
| --- | --- | --- | --- |
| N vs AC | 3.851 | Yes | ** |
| N vs LC | 7.064 | Yes | ** |
| LC vs LC+FS | 10.951 | Yes | *** |
| LC vs LC+FA | 3.882 | Yes | ** |
| LC vs LC+FS+FA | 0.944 | No | ns |

GLUT-1 RT-PCR (Fig. 8A)

| **Bonferroni’s multiple comparison test** | **t** | **Significant? P < 0.05?** | **Summary** |
| --- | --- | --- | --- |
| N vs AC | 2.657 | Yes | * |
| N vs LC | 17.913 | Yes | *** |
| LC vs LC+FS | 16.497 | Yes | *** |
| LC vs LC+FA | 11.474 | Yes | *** |
| LC vs LC+FS+FA | 3.223 | Yes | * |

GLUT-2 RT-PCR (Fig. 8B)

| **Bonferroni’s multiple comparison test** | **t** | **Significant? P < 0.05?** | **Summary** |
| --- | --- | --- | --- |
| N vs AC | 13.716 | Yes | *** |
| N vs LC | 13.740 | Yes | *** |
| LC vs LC+FS | 8.257 | Yes | *** |
| LC vs LC+FA | 4.912 | Yes | ** |
| LC vs LC+FS+FA | 2.889 | Yes | * |
