# Supplementary figures and images for: Mitigating renal dysfunction in liver cirrhosis: Therapeutic role of ferrous sulphate, folic acid, and its co-administration
Source: Toxicol Rep. 2025 Apr 9;14:102026. doi: 10.1016/j.toxrep.2025.102026 (PMC12017912; doi:10.1016/j.toxrep.2025.102026)

## Slide 1
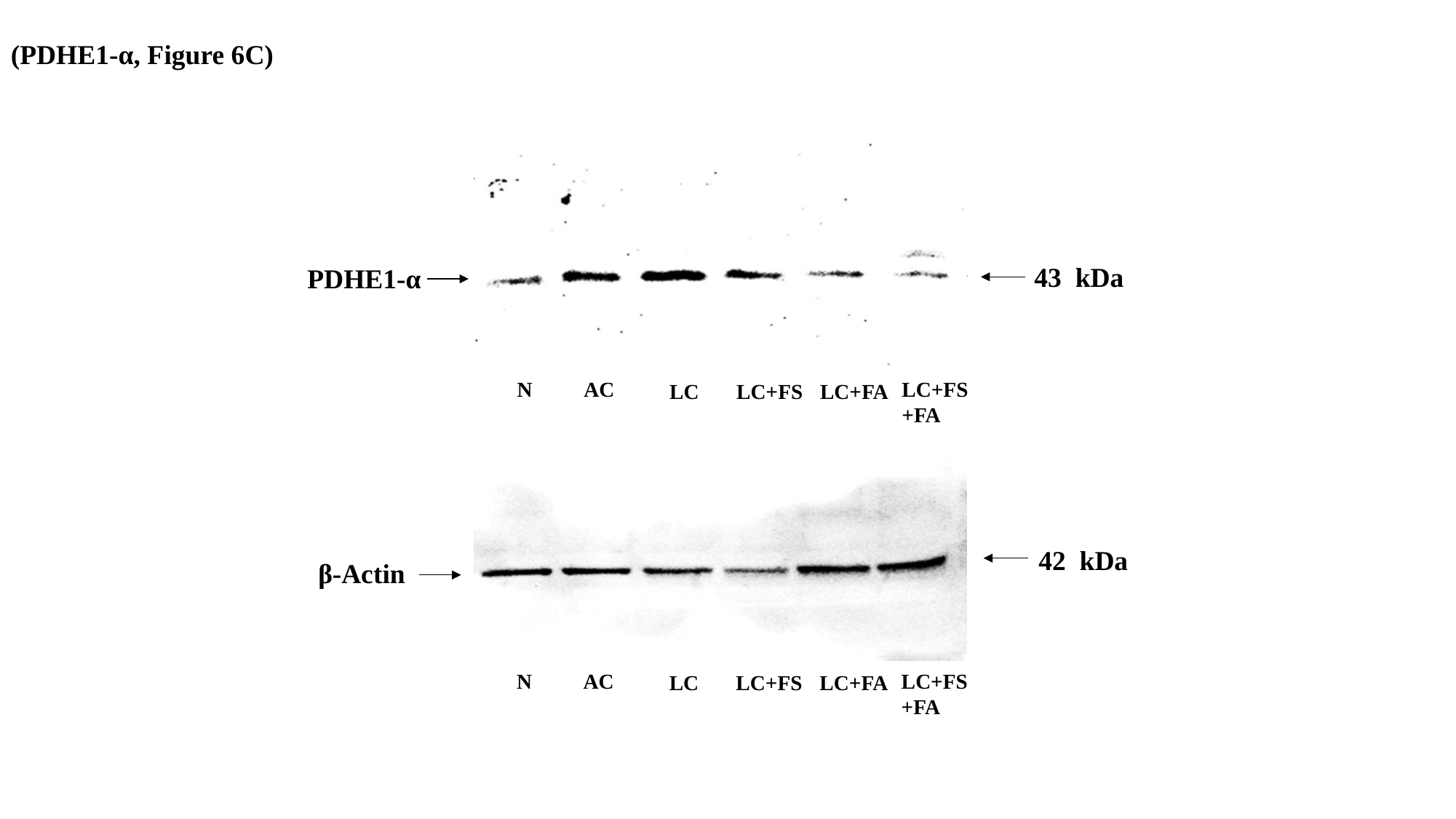

(PDHE1-α, Figure 6C)
43 kDa
PDHE1-α
AC
N
LC+FS
+FA
LC
LC+FS
LC+FA
42 kDa
β-Actin
AC
N
LC+FS
+FA
LC
LC+FS
LC+FA

Supplement: Supplementary file 2 — Supplementary material [file mmc2.pptx]
